# The Role of Teacher-Student Relatedness and Teachers' Engagement on Students' Engagement in EFL Classrooms

**DOI:** 10.3389/fpsyg.2021.745435

**Published:** 2021-08-30

**Authors:** Shiyuan Gan

**Affiliations:** Foreign Language College, Changchun University, Changchun, China

**Keywords:** language learner psychology, teacher-student relatedness, academic engagement, teacher support, language ability enhancement

## Abstract

Relationships in language contexts or interactions between teachers and learners might play an eminent role in EFL learners' language ability development. The current review brings to the fore an overview of teacher-student relationships and factors that contribute to this interaction. It has been revealed that EFL teachers' and learners' expectations, beliefs, personality, knowledge, and the language teaching context all play a role in creating an adequate relationship among teachers and learners. This overview suggests several practical tasks to develop a positive relationship between teachers and learners in EFL classrooms.

## Introduction

Relationship is the key aspect of human social life, and relationship skills are the most important strategies that lead us to success (Lambert and Zhang, 2019; Hiver et al., 2021). According to the self-determination theory (SDT) (Cooper, 2013; Ryan and Deci, 2017), healthy behavior depends on satisfying psychological needs such as autonomy, competence, and relationship. If needs are met to some extent on an ongoing basis, people might grow effectively and function well (Mystkowska-Wiertelak, 2020); nonetheless, if these needs are not met, individuals are more likely to witness abnormalities and dysfunctions (Al-Hoorie, 2016).

Like other learning environments, the most dominant relationship in an EFL [English as a foreign language] learning environment is the relationship between teachers and students (Patall, 2013; Xie and Derakhshan, 2021); therefore, teachers play a significant role in any language teaching-learning process (Derakhshan et al., 2020). Employing this efficient relationship, EFL teachers can facilitate the teaching process and even compensate for textbook deficiencies and lack of facilities (Oppermann and Lazarides, 2021). Conversely, those teachers who lack this ability can turn the best teaching situation and subject matter into an inactive and unattractive learning environment (Phung, 2017). If this connection is established in a language learning environment, educational goals might be achieved with more quality and ease. This interaction is manifested in the relationship between teachers and students. In the teaching process, not only the teachers' experiences and academic approaches but also their whole personalities and methods are effective in creating conditions for students to learn and change (Mouratidis et al., 2013).

One of the factors that play a pivotal role in students' engagement and success in an EFL context is the quality of the teacher-student relationship (Zhang, 2020). This relationship can mediate among students' self-regulation, classroom culture, and academic motivation with students' engagement and success (Froiland and Worrell, 2016; Hiver et al., 2021). From a theoretical point of view, similar to financial poverty that leads to the employment of children or adolescents, motivational poverty also plays an indispensable role in investigating the reason for boredom and dropping out of universities around the world. In other words, language students' outputs do not arise as a result of a simple cause-and-effect relationship, but rather appear to be the result of regular interactions of the individual (teacher and student characteristics) and contextual (educational environment) factors (Derakhshan et al., 2021). Therefore, it can be argued that EFL students' success in educational activities is influenced by their individual differences and the messages they perceive from the language learning context. Close relationships with teachers might promote healthy socio-emotional development and also lead to adaptation and success in language learning environments (Cornelius-White, 2007; Pishghadam et al., 2019). However, language teachers have some challenges such as different levels of parental involvement, decomposing facilities, the risk of violence, and diverse students in their teaching environments (Pennington and Richards, 2016).

It is quite evident that the conventional role of teacher-centeredness in a language teaching context might not be satisfactory to alleviate such growing challenges (Chen and Kent, 2020). A language classroom can be considered as a dynamic context that has its structure and norms and is under the control of subtle behavioral factors (Xiang et al., 2017). Despite its obvious scientific aspect, this relationship is not limited to the transfer of scientific and technical teachings from the teacher to the student (Zhang, 2020). EFL students also perceive behavioral culture, personal character, social perspective, and teaching style. Unlike mechanical relationships, the teacher-student relationship in a language classroom is a complex human relationship in which various factors such as abilities, personality, and family circumstances are in interaction (Xie and Derakhshan, 2021).

Numerous factors attributed to students' emotional arousal might affect the teacher-student relationship and subsequently influence the quality of language teaching and students' language learning enhancements. However, the importance of this relationship has not received especial attention in most EFL contexts. Given the importance of establishing an effective interpersonal relationship in the language teaching process and achieving educational goals in language learning environments, it is necessary to pay more attention to this relationship and help EFL teachers to achieve their basic goal that is, the enhancement of language abilities through an effective relationship.

## Teacher Relatedness and Engagement

EFL researchers have conducted numerous studies and focused on various aspects of the classrooms and teaching contexts to find out the factors that improve the quality of students' educational paths (Reyes et al., 2012; Ryan and Deci, 2017; Dao et al., 2019; Aubrey et al., 2020). Studies have shown that the general policies of any educational system, the perceived social expectation of teacher-student interactions, and teacher-student biological experiences all affect the dimensions of the teacher-student relationship in EFL contexts (Jang et al., 2010). However, this interaction can be positive or negative.

A positive relationship with a teacher in addition to academic achievement (Storch, 2008) will lead to a high level of class participation (Phung, 2017; Pishghadam et al., 2021b), positive academic motivation (Patall et al., 2018), and learners' self-confidence (Mystkowska-Wiertelak, 2020), a feeling of empathy and mutual understanding between teacher and student (Zhang, 2020), and more efficient instructional task design (Lambert and Zhang, 2019). According to Patall (2013), teacher-student relationships are a key part of successful language teaching and learning. Obviously, EFL students need their teachers in many learning environments. A positive teacher-student relationship, motivation, attachment to school, cooperation in class activities, hard work in dealing with problems, friendly help and support, understanding of interpersonal behavior, and creating responsibility and freedom can be effective (Wang et al., 2021). In other words, EFL students who have a warm and intimate relationship with their teachers have high self-confidence, interest in their teacher, more motivation to learn a positive attitude toward school, and enjoy the acceptance of their peers and classmates (Pishghadam et al., 2021a).

In contrast, in a negative relationship, the classroom process can have a moderating effect on solving students' behavioral problems. In other words, students who have inappropriate relationships in the classroom and conflicts with their teachers suffer from problems such as dropping out, being rejected, not being accepted by peers, and increasing inappropriate behaviors. Patall (2013) believes that the educational and emotional interactions of teachers and classmates in students with behavioral problems lead to higher academic achievement and reduction of behavioral problems. It is generally believed that EFL teachers with high self-efficacy are usually more successful in dealing with students with behavioral problems (Oppermann and Lazarides, 2021).

## Academic Engagement

In the last two decades, the concept of students' academic engagement has attracted increasing attention from EFL researchers and educators. Many studies have shown that academic engagement has been able to predict a wide range of developmental and educational outcomes (Reyes et al., 2012). Academic engagement is a multifaceted concept that includes behavioral, cognitive, and emotional (Hiver et al., 2021). According to Patall (2013), academic engagement is considered as a “communication” process that reflects students' cognitive, emotional, behavioral, and motivational capacity and status.

The review of literature has revealed that there is a link between academic engagement and context adjustment. The studies reveal that the quantity and quality of teacher-student interactions significantly predict the level of satisfaction or stress among EFL students (Al-Hoorie, 2016; Wang et al., 2021). They have shown that teacher-student relationships, especially interactions that focus on academic or intellectual content, affect students' academic performance and language ability. They also show that the rate of scientific progress of EFL students who had more interaction with teachers is higher than what was predicted before their enrollment (Dao et al., 2019; Azkarai and Kopinska, 2020; Hiver et al., 2021). Likewise, EFL students who have a negative feeling toward their teacher make less progress than expected. Most students who interact individually with teachers experience the deepest kind of learning; since they had the opportunity to ask questions about their ambiguities and interests (Lambert and Zhang, 2019). Teacher-student interaction includes formal communication within the classroom and informal communication outside the classroom, which is very important for the intellectual development and scientific and educational continuity of students (Oppermann and Lazarides, 2021). Review of related literature has shown that factors such as similarity and closeness between a teacher and students (Azkarai and Kopinska, 2020), teacher's personal relationship with students (Froiland and Worrell, 2016), simplicity and intimacy of a teacher (Hiver et al., 2021), teacher's sense humor (Mystkowska-Wiertelak, 2020), and creating opportunities for asking questions and solving problems (Cooper, 2013) can have a significant impact on creating an effective communication between a teacher and students in the classroom. Looking at an opposite aspect, the researchers stated that interaction between teachers and students outside of the classroom can take many forms: in person or online during office hours, communication via e-mail are examples of these cases. Out-of-class interaction provides teachers with the opportunity to discuss lessons or classroom management styles with students and make them more professional and personal (Patall et al., 2018). Out-of-class communication provides a cycle of extensive and individual interactions, and such opportunities can lead to greater classroom productivity and it will also increase language student learning (Phung, 2017). On the other hand, due to a large number of students, this task may be time-consuming. Communicating with teachers increases the students' self-confidence and motivation to learn (Pennington and Richards, 2016). Considering that the role and expectations of EFL teachers and students in such interactions are not structured; creating a successful relationship requires careful consideration (Pishghadam et al., 2021b).

## Pedagogical Implications

The present findings suggest useful and practical tips and several courses of action to create a positive teacher-student relationship in EFL contexts. EFL teachers should:

### Determine Their Temporal and Spatial Boundaries

Similar to what Patall et al. (2018) argued EFL teachers should think about whether or not they want students to contact their home. Can students visit them outside of office hours? How long should students wait for their email response?

### Identify That These Interactions Are Part of Their Curriculum and Not Random and Purposeless Conversations

These interactions are a part of their teaching responsibilities. It is also important to be aware of the characters in the classroom, both as an EFL teacher and as someone who interacts with students. Similarly, Wang et al. (2021) highlighted the importance of teachers' and learners' personality awareness in language classrooms.

### Be Aware of Their Students' Individual Learning Styles and Teaching Methods

Awareness and attention to different language teaching and learning methods are extremely important; since this knowledge provides a great opportunity to address academic challenges. Many researchers such as Cooper (2013) and Lambert and Zhang (2019) stated that it is essential to notice that the learning method is not the same for all students. They use a wide range of learning methods including competition, collaboration, avoidance, partnership, affiliation, and independence.

### Use Technology to Create Opportunities to Interact With Students

One of the out-of-class communication opportunities that in Phung (2017) words may lead to a greater classroom productivity is electronic mail. Electronic mail can be a convenient way of communicating and provides information between teacher and student outside the classroom, although it creates high expectations and workload, especially in crowded classrooms.

### Evaluate Teacher-Student Interaction

The literature suggests several ways to examine how teacher-student interaction affects language students' learning outcomes. Although what happens in this interaction can be evaluated by qualitative methods, there are simpler and more effective methods for the relative evaluation of these interactions (Storch, 2008; Zhang, 2020). A simple method is to record the number and duration of students' referrals to the teacher and compare it with classroom scores and the tests at the end of the semester. Teachers can also find a list of students in the class by those who come to their office, people who communicate with them online, and students who do not interact, prepare, and observe how each group behaves. They can ask students who interact with them outside the classroom to describe how these interactions affect their learning. Use these experiences to motivate students in future classes.

Further studies need to be done to establish whether educational policies have any impact on teachers' relationship with their students in EFL language classrooms.

## Author Contributions

SG independently read the relavant literature and wrote the paper entitled The Role of Teacher-Student Relatedness and Teachers' Engagement on Students' Engagement in EFL Classrooms and revised it before it was submitted to this special issue.

## Conflict of Interest

The author declares that the research was conducted in the absence of any commercial or financial relationships that could be construed as a potential conflict of interest.

## Publisher's Note

All claims expressed in this article are solely those of the authors and do not necessarily represent those of their affiliated organizations, or those of the publisher, the editors and the reviewers. Any product that may be evaluated in this article, or claim that may be made by its manufacturer, is not guaranteed or endorsed by the publisher.
